# Richter Transformation in Chronic Lymphocytic Leukemia: Update in the Era of Novel Agents

**DOI:** 10.3390/cancers13205141

**Published:** 2021-10-14

**Authors:** Tamar Tadmor, Ilana Levy

**Affiliations:** 1 Hematology Unit, Bnai-Zion Medical Center, Haifa 3339419, Israel; ilana.levy@b-zion.org.il; 2 The Rappaport Faculty of Medicine, Technion-Israel Institute of Technology, Haifa 3525422, Israel

**Keywords:** richter syndrome, richter transformation, chronic lymphocytic leukemia, DLBCL, novel agents, BTKi, BCL2

## Abstract

**Simple Summary:**

Richter transformation is a significant and devastating complication of chronic lymphocytic leukemia. While its pathogenesis has been well-studied in terms of genetic and molecular changes and its diagnosis has been made easier by imaging and pathological techniques, its treatment is still an issue. Most patients are resistant to chemo-immunotherapy, and even novel agents do not seem to improve the prognosis in a significant way. Therefore, new combinations and novel drugs are currently being tested. In the current review, we summarize new data about the pathophysiology, biological, and clinical basis of Richter transformation, as well as the different treatments of this condition.

**Abstract:**

Richter transformation (RT) is a poorly understood complication of chronic lymphocytic leukemia (CLL) with a dismal prognosis. It is associated with a switch in histopathology and biology, generally with a transformation of the original CLL clone to diffuse large B-cell lymphoma (DLBCL) or less frequently to Hodgkin’s variant of Richter transformation (HVRT). It occurs in 2–10% of CLL patients, with an incidence rate of 0.5–1% per year, and may develop in treatment-naïve patients, although it is more common following therapy. In recent years, there has been a deeper understanding of the molecular pathogenesis of RT that involves the inactivation of the TP53 tumor suppressor gene in 50–60% of cases and the activation of aberrations of NOTCH1 and MYC pathways in about 30% of cases. Compared to the preceding CLL, 80% of cases with DLBCL-RT and 30% of HVRT harbor the same IGHV-D-J rearrangements, indicating a clonal evolution of the disease, while the remaining cases represent de novo lymphomas that are clonally unrelated. Despite advances in understanding the molecular variations and the pathogenesis of the disease, there is still no significant improvement in patient outcomes. However, if no clinical trials were designed for patients with RT in the past, now there many studies for these patients that incorporate new drugs and novel combinations that are being explored. In this review, we summarize the new information accumulated on RT with special emphasis on results involving the novel therapy tested for this entity, which represents an unmet clinical need.

## 1. Definition, Epidemiology, and Clinical Presentation of Richter Transformation

Richter transformation (RT) is defined as the occurrence of an aggressive lymphoma in patients with a previous or concomitant diagnosis of chronic lymphocytic leukemia (CLL) [1]. It is characterized by a switch in the histopathology and biology of the original CLL.

In 95–99% of cases, such a switch is towards a diffuse large B cell lymphoma (DLBCL) (DLBCL-RT), cases of the Hodgkin’s variant of Richter transformation (HVRT) (0.5–5%) [2] have been described, and, less frequently, the transformation has been described in plasmablastic lymphomas [3]. 

The exact incidence of the syndrome is unknown, as some cases are probably being missed due to the aggressiveness of the disease or the absence of adequate histopathology samples. In addition, most of the reported series have been of a retrospective nature. Therefore, the reported incidence varies. In the chemo-immunotherapy era, it was reported as 2–10%, with a transformation rate of 0.5–1% per year [4,5,6,7,8,9,10] (Table 1). 

Recently, the incidence of RT was evaluated using the Surveillance, Epidemiology, and End Results (SEER) database of CLL patients diagnosed between 2000 and 2016. In this large cohort of 74,116 patients with CLL, 530 cases with RT were identified, with a 0.7% incidence of transformation [29]. The German CLL Study Group summarized their pooled analysis of 2975 patients included in the frontline treatment trials and an RT incidence of 3% was observed, 92% of which with DLBCL-RT [30]. In the era of novel agents, one raised concern was whether there was an increased rate of this rare and aggressive transformation among patients treated with Bruton tyrosine kinase inhibitors (BTKis) or BCL2 inhibitors (BCL2is). Indeed, in the first clinical trials using novel agents, 2–15% incidence rates of RT have been described in relapsed/refractory (R/R) patients with CLL treated with ibrutinib [11,12,13,14,31,32], venetoclax [18,19,20], or idelalisib [15,16,17]. These alarming reports were probably related to the recruitment of patients with R/R disease or even already in the early stages of transformation (Table 1).

In contrast, in clinical trials involving treatment-naïve patients with CLL treated with novel agents, the incidence of RT was reported to be 0–4% [14,21,22,23,24,25,26,27,28] (Table 1), indicating that there is no increase in the number of cases of RT during therapy with these novel and effective biological agents.

Clinical suspicion of RT should be raised when a patient with CLL presents with the sudden clinical deterioration with prominent constitutional symptoms, including fever, night sweats, and loss of weight. On physical examination, there is an asymmetric and rapid growth of bulky lymph nodes or extra nodal involvement that is characteristic in 40% of all RT cases, mostly those of the gastrointestinal tract, bone marrow, central nervous system, and skin [33,34]. Laboratory tests frequently present with cytopenias, an elevation of lactate dehydrogenase (LDH), and (less frequently) with hypercalcemia [35].

## 2. Pathogenesis and Risk Factors for the Development of Richter Transformation

It is of great interest to identify the patients with the highest risk to develop RT. Indeed, risk factors for the development of RT have been extensively studied and include clinical characteristics or molecular and genetic changes.

Clinical risk factors for RT include: bulky lymphadenopathy or hepato-splenomegaly, advanced stage, low platelet count, elevated beta-2-microglobulin [5,33,36,37], past CLL therapy combining purine analogues and alkylating agents, and a higher number of lines of therapy [38]. These clinical observations are probably only surrogate aspects secondary to some intrinsic biologic features of an individual tumor.

Of major interest are the molecular mechanisms that favor the transformation of CLL into lymphoma. In this review, we chose to summarize these pathways based on the stage at which they occur in CLL or RT diagnosis.

### 2.1. Molecular and Genetic Changes at CLL Diagnosis Associated with Richter Transformation

The genetic background represented by single nucleotide polymorphism (SNP) analysis may be of interest with selected germline SNPs that may confer susceptibility to RT [39]. One such observation was the report that GG homozygosity for the rs6449182 SNP of CD38 gene encoding appears to be associated with an increased risk of RT. Similarly, patients with BCL2 GG and LRP4 TT germline genotypes seem to have a higher risk of developing RT [40,41].

Studies of the immunoglobulin heavy-chain variable region gene (IGHV) mutational status indicate that patients with unmutated IGHV [40] or stereotyped B-cell receptor (BCR) [5,33,40,41,42,43] have increased risk of RT. Moreover, IGHV4–39 gene usage has been shown to carry a 24-fold increased risk of RT and when combined with stereotyped BCR (SUBSET 8) in the same patient, it showed a 5 year risk of RT of 68.7% [6]. Another recently noted point is that CLL patients with a complex karyotype at diagnosis seem to have the highest risk and shortest time to Richter transformation [44,45].

Genomic aberrations at CLL diagnosis that increase the risk of RT include CDKN2A deletion, TP53 disruption, C-MYC activation, trisomy 12 (particularly in the absence of del13q14), and NOTCH1 mutation [38]. Previously, two mechanisms for the occurrence of RT have been described. The main mechanism, which is found in about half of patients with RT, is related to the inactivation of TP53 and of CDKN2A/B [46], which induces cell cycle deregulation. The second reported pathway is defined by the presence of trisomy 12 or NOTCH1 mutations present in about one third of cases [46]. Indeed, the mutational status of NOTCH1 has been reported in 45% of cases with transformation into DLBCL compared to CLL without NOTCH1 mutations, in which only 4% have undergone RT [47].

Murine models have shown that RT is characterized by constitutive active AKT, which seems to induce NOTCH1-signaling B cells via the NOTCH1 ligand expressed by T cells, and therefore apparently orchestrates RT [48]. 

### 2.2. Molecular and Genetic Changes Characterizing Richter Transformation

Other molecular and genetic markers have been analyzed from the point in time of the documentation of RT. Mutational profile and ex vivo pathway analyses from patient-derived xenograft models obtained from two RT patients’ samples engrafted into immunocompromised mice revealed the overactivation of the BCR, NFkB, and NOTCH pathways, while RNA sequencing showed that more than 80% of the transcriptome was shared between primary CLL and RT xenograft [49]. A stable RT cell line established from the cervical lymph node of a 60-year-old patient with CLL and clonally-related RT-DLBCL revealed a complex karyotype with the loss of TP53 and CDKN2A, a chromosomal gain of the NOTCH1 gene locus, and strong immunoreactivity for BCL-2 [50]. Furthermore, the whole-genome sequencing and RNA expression data of paired circulating CLL and RT biopsies performed in 17 patients with CLL identified a high number of mutations in poor-risk CLL drivers and genes in the DNA damage response pathway (that seems to be the dominant mechanism driving RT), as well as other genomic aberrations including the protein tyrosine phosphatase receptor and tumor necrosis factor receptor-associated factor 3 in RT biopsies [51]. Moreover, the noncoding genome of RT biopsies revealed mutations affecting the regulatory regions of key immune-regulatory genes (BTG2, CXCR4, NFATC1, PAX5, NOTCH-1, SLC44A5, FCRL3, SELL, TNIP2, and TRIM13) [51]. In the novel therapy era, new genetically and molecularly defined RT have seemed to appear [52]. For example, two cases of post-ibrutinib RT have been reported and were shown to lack resistance mutations of the BTK and PLCG2 genes, which are clonally related to the preexisting CLL phase [35]. Finally, downregulating cell cycle inhibitors (e.g., inactivating lesions in CDKN2A, CDKN2B, and TP53) have been associated with RT, and the BCR stimulation of human RT cells containing such lesions seems to induce proliferation [53]. Therefore, RT seems to have a unique genomic and molecular expression that appears to impact its pathogenesis, as well as prognosis, since TP53 abnormalities and IGHV unmutated status at both CLL diagnosis and the time of RT seem to be associated with poor prognosis in RT patients [54].

### 2.3. Microenvironment

It is understood that the microenvironment has a fundamental role in the supporting cancer genesis. CLL cells and their surrounding niche are closely related and constantly interact. From this point of view, microenvironment remodeling also seems to have a role in the development of RT. This observation is reflected by a high programmed death 1 (PD-1) expression by tumoral B lymphocytes [55,56], higher programmed death ligand 1 (PD-L1) expression in histiocytes and dendritic cells, the higher infiltration of FOXP3-positive T cells and CD163-positive macrophages, and lower peripheral blood T-cell receptor clonality compared to CLL without RT [56], suggesting changes in the immune signature of CLL after RT.

## 3. Diagnosis of Richter Transformation

It is highly important to have a high index of suspicion of RT in a CLL patient with sudden clinical deterioration and to direct them to workout with the aim of performing a biopsy from the most accurate site for diagnosis as early as possible.

### 3.1. Pathological Diagnosis

The diagnosis of RT is based on a biopsy and the histopathologic analysis of a suspected lesion (mainly lymph node) by an expert haemato-pathologist. 

Such a diagnosis of DLBCL-RT is still a pathologic challenge due to the difficulty of differentiating DLBCL-RT from “accelerated CLL” or de-novo DLBCL [57]. 

A tissue sample is typically infiltrated by large neoplastic B-lymphocytes with nuclear size equal or larger than macrophage nuclei or more than twice a normal lymphocyte, with a morphology similar to centroblasts in 60–80% of cases or immunoblasts in 20–40 of all cases [57].

The cell of origin is generally of an activated-B-cell (ABC) type that expresses post-germinal center markers such as IRF-4, whereas only 5–10% display a germinal center B-cell (GCB) phenotype expressing CD10 and/or BCL6 [58]. Moreover, CLL markers such as CD5 and CD23 are generally lost during RT [59]. Due to the complexity of distinguishing DLBCL-RT from histologically aggressive CLL, criteria for the histological diagnosis of DLBCL-RT have been delineated [57].

Clonal relationship analysis: An important step following the histopathologic diagnosis of RT is to perform an analysis of the clonal relationship of the RT tissue biopsy, as results have both prognostic and therapeutic implications. IGHV-D-JH nucleotide rearrangement should be sequenced by PCR or next-generation sequencing (NGS) methods, and results should be compared with those of circulating B-CLL cells.

**Figure 1:**
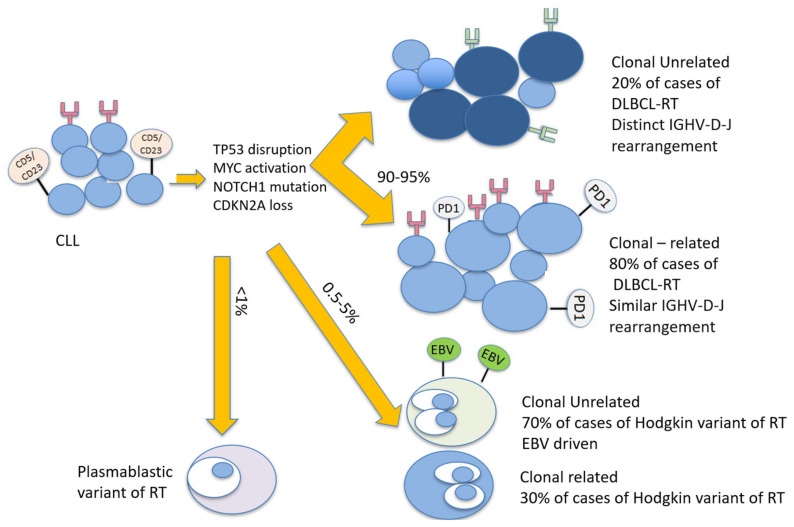
Biological pathways associated with Richter transformation. Legend: CDKN2A: cyclin-dependent kinase inhibitor 2A; CLL: chronic lymphocytic leukemia; DLBCL-RT: diffuse large B cell lymphoma Richter transformation; IGHV-D-J: immunoglobulin heavy chain variable D-J; TP53: tumor protein 53.

Clonally related transformation occurs in 80% of all cases of RT and represents “true transformation” with a dismal outcome, chemotherapy resistance, and a high expression of PD1, while “clonally unrelated” RT has shown similar outcomes to de novo DLBCL (Figure 1).

### 3.2. Radiological Diagnosis

**Figure 2:**
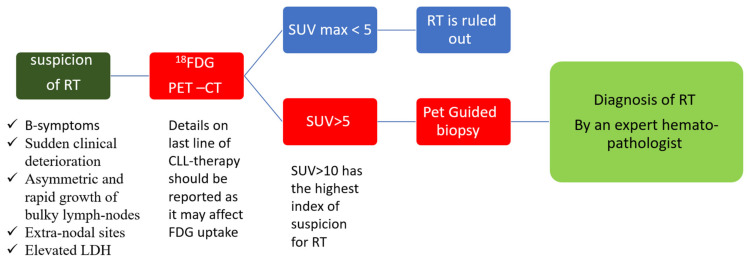
Diagnosis of Richter transformation. Legend: 18-FDG-PET–CT: positron emission tomography with 2-deoxy-fluorine-18-fluoro-D-glucose; CLL: chronic lymphocytic leukemia; LDH: lactate dehydrogenase; RT: Richter transformation; SUV: standardized uptake values.

As opposed to CLL, radiological evaluation is recommended for the workup diagnosis of RT. Conventional CT has been performed in the past, but it currently has limited use and is only recommended if other imaging modalities are not available [60]. 18-FDG-PET/CT is the recommended imaging technique, both for diagnosis and as a guide for the most adequate site of accurate biopsy. The probability of RT was shown to be significantly increased with higher standardized uptake values (SUVs) and maximal SUV (SUV_max_). This technique has the ability to distinguish between CLL (median SUV_max_: 3.7), accelerated CLL (median SUV_max_: 6.8), and RT (median SUV_max_: 17.6) [61]. Therefore, an 18-FDG-PET/CT showing low uptake (SUV of 5 and lower) seems to rule out RT, while an 18-FDG-PET/CT showing a high uptake (SUV of 10 and higher) may help guide biopsies for definite RT diagnosis [62,63,64] (Figure 2). Furthermore, some 18-FDG-PET/CT markers such as total metabolic tumor volume, SUV body weight, SUV lean body mass, SUV body surface area, lesion-to-liver SUV ratio, and lesion-to-blood-pool SUV ratio assessed at the time of RT into DLBCL seem to be correlated with overall survival (OS) [65,66]. However, in the novel therapy era, one should consider the limitations of 18-FDG-PET/CT, the results of which might be influenced by the use of biological agents as BTKi and anti-PD1 [67]. Other imaging techniques involving novel PET radiotracers, whole-body diffusion-weighted imaging, radiomics, and PET–MRI seem promising in this area [67].

## 4. Current Treatment Strategies of Richter Transformation

### 4.1. Chemo-Immunotherapy

**Figure 3:**
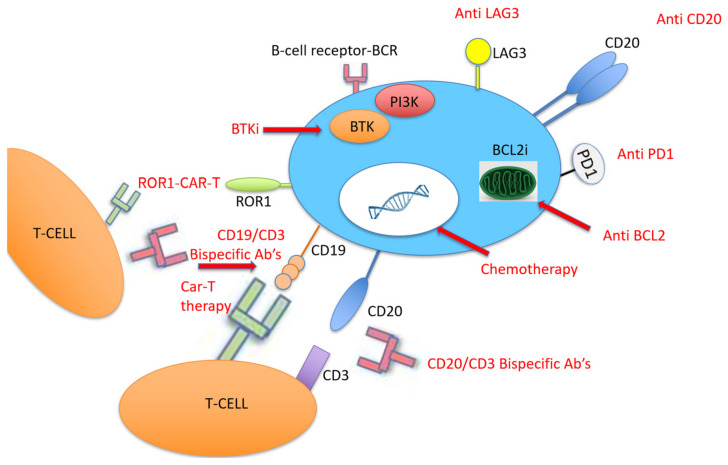
Target therapy and chemo-immunotherapy for the treatment of Richter transformation. Legend: Abs: antibodies; BCL2: B-cell lymphoma 2; BCL2i: B-cell lymphoma 2 inhibitor; BTK: Bruton tyrosine kinase; BTKi: Bruton tyrosine kinase inhibitor; CAR-T: chimeric antigen receptor T cell; LAG3: lymphocyte-activating 3; PD1: programmed death 1; PI3K: phosphoinositide 3-kinase; ROR1: receptor tyrosine kinase-like orphan receptor 1.

Various chemotherapy and chemo-immunotherapy protocols have been tested for DLBCL-RT, including OFAR-1 [68], OFAR-2 [69], R-CHOP [70], O-CHOP [71], R-Hyper-CVAD [69], R-EPOCH [72], DHAP/ESHAP [73], Hyper-CVXD [74], and R-Hyper-CVXD [75] (Figure 3). In the chemotherapy era, the median OS from time of diagnosis of clonally related RT was less than a year [4,76]. The addition of rituximab to chemotherapy for RT improved the 2 year OS from 19% in the chemotherapy alone arm to 42% [76] (Table 2). However, even though the use of chemo-immunotherapy for RT has achieved unsatisfactory results, it remains the gold standard therapy outside clinical trials.

Eligible patients who are chemo-sensitive and achieve good response following chemo-immunotherapy are recommended to undergo allogeneic stem cell transplant.

### 4.2. Stem Cell Transplantation

Stem cell transplant (SCT) represents the only option for curing RT. The European bone marrow transplantation registry included 59 patients with RT from 1997 to 2007: 34 and 25 of them underwent autologous SCT and SCT, respectively, most of them with reduced intensity conditioning (RIC) [77]. The 3 year OS was estimated at 36% for allogeneic SCT and 59% for autologous SCT, with an age younger than 60 years, chemo-sensitive disease, and RIC being associated with a better prognosis after allogeneic SCT in RT [77]. The benefits of RIC preceding allogeneic SCT in RT were also underlined in a recent retrospective study including 58 CLL patients, 23 of them with RT with a median follow-up of 68 months that revealed a 5 year OS of 58% and a 5 year PFS of 40% [78]. Another single center study showed encouraging results in 10 patients with RT referred to allogeneic SCT after objective response to therapy, with a 4 year OS of 50%, a non-relapse mortality at both 1 and 4 years post-transplantation of 40%, and a 4 year incidence of relapse/progression of 10% [79]. A recent study included 27 patients with DLBCL-RT and one with HVRT, showing 4 year OS and PFS of 53% and 39%, respectively, and an acceptable 18% rate of grade III–IV graft-versus-host disease [80]. Finally, a systematic review and meta-analysis of four studies including 72 fit patients with RT that underwent allogeneic SCT identified an encouraging pooled overall response rate (ORR), complete remission (CR), OS, and PFS rates of 79%, 33%, 49%, and 30%, respectively [81].

### 4.3. Novel CLL Therapies for RT

With the advent of new drugs that have entered into use in CLL, it was expected that the next step would be to test their effectiveness in RT (Figure 3). The BTKi ibrutinib has been evaluated as monotherapy in eight patients with DLBCL-RT: one of them achieved CR lasting for 2.8 months and three achieved partial remission (PR) lasting between 8 and more than 12 months [82,83]. In three other patients with RT, ibrutinib has been tested in combination with ofatumumab: one of them achieved PR lasting for 4.6 months [84]. The novel BTKi acalabrutinib was tested in 25 patients with DLBCL-RT, 48% of them with prior ibrutinib treatment, and showed a median PFS of 2.1 months [85]. Acalabrutinib is currently tested in combination with six courses of R-CHOP followed by acalabrutinib maintenance in newly diagnosed RT [86]. Concerning BCL2i, venetoclax monotherapy was tested in seven patients with DLBCL-RT, and three of them (43%) achieved PR [87]. Real-world analysis from a French compassionate use venetoclax program including seven RT patients treated with venetoclax, most of them with complex karyotype, showed an ORR of 29% (2/7) and a median OS of only 1.1 months [88]. When combined with chemo-immunotherapy (R-EPOCH) in 27 patients with RT, venetoclax showed a 48% CR, a 11% PR, and a median PFS and OS of 16.3 months both [89] (Table 3). Therefore, considering the better outcome of novel agents combined with chemo-immunotherapy, a recent review suggested a synergistic effect of these approaches [90].

### 4.4. PD-1/PD-L1 Pathway

Due to the relatively high expression of PD-1 and PD-L1 in DLBCL-RT compared to de novo DLBCL [101], therapy with PD-1 monoclonal antibodies (PDCD1) has been tested in patients with RT, all of them with prior BTKi therapy (Figure 3). PDCD1 was given as monotherapy or combined with ibrutinib with or without venetoclax. Only one patient responded, and the median OS was 2 months [91]. More recently, checkpoint inhibitors were tested in patients with DLBCL-RT. Nine patients with DLBCL-RT were treated with pembrolizumab monotherapy with a 44% ORR [92], and 23 patients received nivolumab combined with ibrutinib with an ORR of 43% [94]. Another study recently evaluated the effect of pembrolizumab on 23 patients with R/R RT, showing an ORR of 13% (3 patients), although two of them had Hodgkin’s lymphoma histology [93] (Table 3). Following this potential clinical activity of checkpoint inhibitors in DLBCL-RT, some clinical trials were recently initiated. A German CLL study group is currently recruiting RT patients to assess the efficacy and safety of the BTKi zanubrutinib combined with the PD-1 inhibitor tislelizumab for the CLL-RT1 study (NCT04271956), and the United States CLL study group has started recruiting for the Pembro-U2 phase I/II clinical trial aiming to assess the safety and efficacy of U2 (both anti-CD20 ublituximab and anti-phosphoinositide 3 kinase (PI3K) umbralisib) combined with the anti-PD1 pembrolizumab in patients with R/R CLL and RT (NCT02535286).

### 4.5. Bispecific Monoclonal Antibodies

Bispecific monoclonal antibodies have recently been adopted for the treatment of different lymphoproliferative disorders such as acute lymphoblastic leukemia, multiple myeloma, and DLBCL [102]. Alderuccio et al. recently described a case of refractory RT with rapid CR following therapy, and they found that the bispecific anti-CD19/anti-CD3 monoclonal antibody blinatumomab permitted bridging to allogeneic SCT (Figure 3) [95]. Following this success, the MD Anderson CLL study teams designed a phase II open-label clinical trial aiming to test the efficacy and safety of this drug in RT (NCT03121534).

### 4.6. Chimeric Antigen Receptor T Cell Therapy

Another approach that was recently adopted for the treatment of hematological malignancy with promising results is CD19-targeted chimeric antigen receptor T (CAR-T) cell therapy [103]. CAR-T therapy was evaluated in 24 patients with high-risk, heavily pretreated R/R CLL after ibrutinib failure, five of them with RT, and an ORR of 71% was reported at 4 weeks after CAR-T cell infusion [96]. The same study group then tested this therapy among 19 patients concurrently treated with ibrutinib, four of them with RT; they found a 4 week ORR of 83%, a 61% minimal residual disease (MRD) negativity, and 1 year OS and PFS of 86% and 59%, respectively [97]. Due to these encouraging results, a recent Israeli study included eight patients with high-risk CLL with RT that were treated with CAR-T cell therapy in the 2019–2020 period; they reported a 71% (5/8) ORR, that all of them achieved CR on day 28, and a reasonable safety profile of seven patients with cytokine release syndrome—four of them were grade 1, three patients had neurotoxicity, and there were no CAR-T-cell-related fatalities [98]. Another recent group studied nine patients with RT treated with axicabtagene ciloleucel CAR-T cell therapy in a single center in Ohio; eight of them underwent formal response assessment and achieved an objective response (five cases of CR and three cases of PR as the best responses) [99] (Figure 3).

### 4.7. Innovations and Future Directions

Combining the mechanisms of action of novel therapies represents the future for effective therapy in RT. One example is the study of the “synthetic lethality” approach, which was recently investigated by Mato et al., who combined a triplet of a novel and clinically differentiated irreversible BTKi (DTRM-12) with the mechanistic target of rapamycin (mTOR) inhibitor everolimus and the immune-modulator pomalidomide to form an optimized, oral, once-daily DTRM-55. The study included 13 patients with RT-DLBCL and 11 with R/R DLBCL, and the 11 evaluable RT patient had an ORR of 45% and a median duration of response of 15 months [100] (Table 3).

Other combinations currently being tested include the PI3K-inhibitor duvelisib combined with the BCL2i venetoclax in a recruiting phase I/II study on R/R CLL and RT (NCT03534323) and the anti-CD20 monoclonal antibody obinutuzumab with the BTKi ibrutinib and the BCL2i venetoclax in an Israeli phase II study (NCT04939363), but results are still pending.

Moreover, xenograft research is currently being tested as a model for in vivo efficacy for RT. VLS-101, an antibody–drug conjugate targeting receptor tyrosine kinase-like orphan receptor 1 (ROR1)-expressing cancers, has been studied in four RT patient-derived xenografts with varying levels of ROR1 expression, showing CR in those with higher levels of ROR1 expression [104]. This approach is currently being tested in a phase 1 clinical trial in patients with RT and other hematological malignancies (NCT03833180). Another new combination to be considered is co-treatment with the bromodomain extra-terminal (BET) inhibitor or BET-PROTAC and ibrutinib or venetoclax, which has already shown a synergistic in vitro effect in RT cells [105].

## 5. Conclusions

In conclusion, RT remains a rare clinical occurrence. The increasing understanding of the molecular mechanisms underlying this syndrome and of the relevant risk factors may help clinicians to identify high-risk patients with CLL. However, despite the promising primary results of bispecific antibodies and CAR-T cells, the treatment of RT is still an unmet clinical need, and current data on treatment approaches have mainly been derived from small non-randomized trials.

## Figures and Tables

**Table 1:** Epidemiology of Richter transformation reported in clinical trials in the era of chemoimmunotherapy and novel agents.

Trial Reference	Treatment	Del (17p)	Incidence of Richter Transformation
Chemo-immunotherapy			
Tsimberidou, 2006 [4]	Chemo-immunotherapy	NA	3.7%
Parikh, 2013 [5]	Chemo-immunotherapy	3.3%	2.3%
Robak, 2004 [9]	Cladribine, Alkylating	NA	0.9%
Rossi, 2009 [6]	Chemo-immunotherapy	NA	8.8%
Catvosky, 2007 [7]	F vs. FC vs. Chl	NA	1.7%
Solh, 2013 [8]	F vs. Chl vs. F and Chl	NA	6.5%
Fischer, 2016 [10]	FC vs. FCR	6.2%	4.0%
Novel therapies—R/R CLL			
Munir, 2019 [11]	Ibrutinib	32%	10%
O’Brien, 2016 [12]	Ibrutinib	100%	12%
Chanan-Khan, 2016 [13]	Ibrutinib and BR	0	0
Ahn, 2017 [14]	Ibrutinib	60%	9%
Furman, 2014 [15]	Idelalisib and R	42%	NA
Jones, 2017 [16]	Idelalisib and O	40%	NA
Zelenetz, 2017 [17]	Idelalisib and BR	33%	2%
Roberts, 2017 [18]	Venetoclax	30%	16%
Stilgenbauer, 2016 [19]	Venetoclax	100%	10%
Seymour, 2017 [20]	Venetoclax and R	31%	10%
Novel therapies—Treatment naive CLL			
Burger, 2015 [21]	Ibrutinib	0	0
Ahn, 2017 [14]	Ibrutinib	60%	4%
Woyach, 2018 [22]	Ibrutinib Ibrutinib and R	5% 8%	0 1%
Moreno, 2019 [23]	Ibrutinib and O	12%	0.9%
Shanafelt, 2019 [24]	Ibrutinib and R	0.6%	NA
Sharman, 2020 [25]	Acalabrutinib Acalabrutinib and O	8.9% 9.5%	3% 1%
O’Brien, 2015 [26]	Idelalisib and R	14%	0
Lampson, 2019 [27]	Idelalisib and O	17%	NA
Fischer, 2019 [28]	Venetoclax and O	12%	1%

Legend: B: bendamustine; C: cyclophosphamide; Chl: chlorambucil; CLL: chronic lymphocytic leukemia; F: fludarabine; NA: Not assessed; O: Obinutuzumab; R: Rituximab.

**Table 2:** Chemo-immunotherapy outcomes in the treatment of RT.

Regimen	Author, Year	Institution	No. of Patients	Median Age (Years)	CR (%)	ORR (%)	Median PFS (mo)	Median OS (mo)
OFAR-2	Tsimberidou, 2013 [69]	MDACC	35	63	6	39	3	7
OFAR-1	Tsimberidou, 2008 [68]	MDACC	20	66	20	50	4	8
R-CHOP	Langerbeins, 2014 [70]	GCLLSG	15	69	7	67	10	21
O-CHOP	Eyre, 2016 [71]	UK	37	66	25	44	6	11
R-Hyper-CVAD	Tsimberidou, 2013 [69]	MDACC	35	NA	NA	46	6	9
R-EPOCH	Rogers, 2018 [72]	OSU	46	67	20	38	4	6
DHAP, ESHAP	Durot, 2015 [73]	France	28	63	25	43	7	8
R-Hyper-CVXD	Tsimberidou, 2003 [75]	MDACC	30	59	27	43	6	8
Hyper-CVXD	Dabaja, 2001 [74]	MDACC	29	61	38	41	NA	10

Legend: CR: complete remission; mo: months; No: number; ORR: overall response rate; OS: overall survival; PFS: progression-free survival.

**Table 3:** Novel agent evaluated for the treatment of RT.

Regimen	Author, Year	Institution	No. of Pts	Median Age (yrs)	CR (%)	ORR (%)	Median PFS (mo)	Median OS (mo)
Ibrutinib	Tsang, 2015 [82]	Mayo	4	67	50	75	NA	NA
Ibrutinib	Visentin, 2019 [83]	Italy	4	69	0	25	NA	NA
Ibrutinib and O	Jaglowski, 2015 [84]	Ohio	3	64	0	33	NA	NA
Acalabrutinib	Hillmen, 2016 [85]	San Diego	25	NA	9.5	38	2.1	NA
Veneto	Davids, 2017 [87]	Dana-Farber	7	73	0	43	1	6
Veneto	Bouclet, 2021 [88]	France	7	67	0	29	NA	1.1
Veneto and R-EPOCH	Davids, 2020 [89]	Dana-Farber	27	63	48	59	16.3	16.3
PDCD1	Rogers, 2019 [91]	Ohio	10	69	10	10	NA	2
Pembro	Ding, 2017 [92]	Mayo	9	69	11	44	5.4	10.7
Pembro	Armand, 2020 [93]	Dana-Farber	23	NA	4.3	13	1.6	3.8
Nivo and Ibru	Jain, 2016 [94]	MDACC	23	65	35	43	NA	13.8
Bispecific	Alderuccio, 2019 [95]	Italy	1	NA	0	100	NA	NA
CAR-T	Turtle, 2017 [96]	Hutchinson	5	65	NA	71	NA	NA
CAR-T and Ibru	Gauthier, 2020 [97]	Hutchinson	4	65	NA	83	NA	NA
CAR-T	Benjamini, 2020 [98]	Israel	8	64	71	71	NA	NA
CAR-T	Kittai, 2020 [99]	Ohio	8	64	62	100	NA	NA
DTRM-55	Mato, 2020 [100]	Memorial Sloan	13	71	NA	45	NA	NA

Legend: CR: complete remission; Ibru: ibrutinib; mo: months; Nivo: nivolumab; No: number; O: ofatumumab; ORR: overall response rate; OS: overall survival; Pembro: pembrolizumab; PFS: progression-free survival.
